# Base editing restores CDKL5 expression and rescues neuronal deficits in a patient-derived model of CDKL5 deficiency disorder

**DOI:** 10.1038/s41598-026-48097-y

**Published:** 2026-04-10

**Authors:** Yue Chai, Yao Zhu, Jiayi Zhu, Mingfeng Guan, Zhongyu Zheng, Yu Chen, Hayley Wing Sum Tsang, Tao Ye, Jacque Pak Kan Ip

**Affiliations:** 1 School of Biomedical Sciences, The Chinese University of Hong Kong, Hong Kong, SAR China; 2 Shenzhen Key Laboratory of Translational Research for Brain Diseases, The Brain Cognition and Brain Disease Institute, Shenzhen Institutes of Advanced Technology, Chinese Academy of Sciences, Shenzhen-Hong Kong Institute of Brain Science—Shenzhen Fundamental Research Institutions, Shenzhen, China; 3 Guangdong Provincial Key Laboratory of Brain Science, Disease and Drug Development, HKUST Shenzhen Research Institute, Shenzhen, China; 4 Division of Life Science, The Hong Kong University of Science and Technology, Hong Kong, SAR China; 5 CUHK Shenzhen Research Institute, The Chinese University of Hong Kong, Shenzhen, China; 6 Gerald Choa Neuroscience Institute, The Chinese University of Hong Kong, Hong Kong, SAR China

**Keywords:** Neurodevelopmental disorder, Cyclin-dependent kinase like 5 (*CDKL5*), CDKL5 deficiency disorder, iPSCs, Adenine base editing (ABE), Gene correction, Neuroscience, Stem cells

## Abstract

Cyclin-dependent kinase like 5 (CDKL5) deficiency disorder (CDD) is a rare monogenic neurodevelopmental disorder caused by pathogenic mutations in the *CDKL5* gene, with approximately 50% of reported variants being point mutations. Base editing presents a promising therapeutic strategy to correct such mutations, restore endogenous CDKL5 expression, and pave the way for novel treatments for CDD. To assess the therapeutic potential of base editing for CDD, we applied adenine base editing (ABE) to correct a *CDKL5*-R550* (c.1648 C > T) mutation in induced pluripotent stem cells (iPSCs) derived from a CDD patient. Isogenic control, *CDKL5*-R550* mutant, and ABE-corrected iPSCs were differentiated into neurons and the restoration of CDKL5-related and functional recovery were assessed. In this study, we demonstrated that ABE successfully restored CDKL5 protein levels and CDKL5-dependent signalling pathways in edited iPSC-differentiated neurons to levels comparable to the isogenic control. Morphological deficits, and genes expression were normalized in the ABE-corrected neurons. This study provides evidence that ABE can precisely correct pathogenic mutation and functionally rescue some CDD-associated neuronal phenotypes in patient-derived cells, supporting its potential as a valuable gene therapy for CDD. Moreover, these findings underscore the broader applicability of base editing for treating other monogenic neurodevelopmental disorders caused by point mutations.

**Supplementary Information:**

The online version contains supplementary material available at 10.1038/s41598-026-48097-y.

## Introduction

Neurodevelopmental disorders (NDDs) affect more than 3% of children worldwide^1^. Despite the diverse genetic causes of these disorders, patients often present with overlapping comorbidities, including intellectual disability, autistic features, visual impairment, and motor abnormalities^2^. Monogenic NDDs, caused by mutations in a single gene, provide clear targets for studying disease pathogenesis and developing targeted therapeutics.

CDD is a rare monogenic NDD, with an estimated prevalence of 1/40,000–1/60,000 live births^3^. It is characterized by early-onset seizures, severe intellectual disability, gross motor impairment, and cortical visual impairment^3,4^. Currently, there is no effective treatment for CDD, and lifelong clinical management is needed. CDD is caused by mutations in *CDKL5* gene^5–7^, which encodes a Cyclin-dependent kinase like 5, and plays multiple roles in the regulation of neuronal development, dendritic morphogenesis and synapse formation^8^. The CDKL5 variant database has identified hundreds of mutations, nearly 50% of which are point mutations^7^. Emerging evidence suggests that CDD may be treatable by genetic manipulation^9^ or gene replacement^10,11^. Furthermore, different *CDKL5* isoforms may have distinct neuronal functions and subject to endogenous regulation^10^. Given the lack of effective therapies of *CDKL5* deficiency in the human brain^12–15^, the development of precise gene therapy for CDD treatment is both urgent and essential.

Base editors are CRISPR-based (clustered regularly interspaced short palindromic repeats) genome-editing tools that can precisely correct point mutations with high efficiency and low incidence of off-target events^16^. Although point mutations account for a significant proportion of pathogenic variants in *CDKL5*, no therapeutic studies have yet explored the application of base editing for CDD, and its application for NDDs remains largely unexplored. To explore the therapeutic potential of base editing in CDD, we focused on correcting the *CDKL5*-R550* mutation (c.1648 C > T, p.R550*), a pathogenic variant initially identified in a female patient with severe mental retardation, early-onset seizures and Rett-like features^17^, and has since been reported in additional cases^18,19^. The *CDKL5*-R550* mutation, located in exon 12, is caused by a transition of cytosine to thymine (C-to-T), generating a premature termination codon (PTC) in the large C-terminus of CDKL5 and leading to the absence of the protein^17^. In addition, this mutation is particularly amenable to the correction by adenine base editing (ABE), a technology capable of converting an A•T base pair to a G•C base pair^16,20^. This approach offers a promising strategy to restore functional *CDKL5* expression and address the underlying genetic cause of CDD.

Patient-derived iPSCs serve as a powerful cellular model to understand the molecular mechanism of CDD and to develop therapeutics^21^. CDKL5 is highly expressed in neurons and plays a crucial role in the neuronal development and morphogenesis^8,22^. For instance, quantitative proteomics identified mitochondrial dysfunction in *CDKL5* p.R59* iPSC-induced neurons (iNs)^23^. In addition, proteomic and phosphoproteomic analyses of CDD patient-derived iN cells have revealed disruption of several signalling pathways, including those regulating microtubule-based cytoskeleton organization^24^. Although gene replacement therapy has shown therapeutic effects in *Cdkl5* knockout mice, different *CDKL5* isoforms have been found to rescue distinct aspects of neuronal morphology and function in iN cells^10^. These previous reports showed that CDD patient-derived iPSC models possess the pathogenic background for the study of neuronal pathogenesis and development of therapeutics. *CDKL5*-R550* and Control iPSCs in this study are isogenic and generated from the same female CDD patient with different inactivation status of the X chromosome^25^, so this allows us to compare mutant-specific defects in the same genetic background. However, it remains unclear whether the impaired neuronal morphology and function in iN cells derived from CDD patient iPSCs can be effectively rescued by base editing. More importantly, patient-derived iPSCs and iN cells models are essential for preclinical studies before base editing can be translated into clinical treatment of CDD.

In this study, we present the first therapeutic application of base editing for CDD, and demonstrated that ABE can precisely correct the pathogenic CDKL5 c.1648 C > T mutation and functionally ameliorated CDD-associated deficits compared to the *CDKL5*-R550* and its isogenic control iN cells. This ABE gene correction strategy restores physiological CDKL5 expression while preserving native regulation of its different isoforms in the brain—an important consideration for the development of gene therapies for CDD and many other NDDs.

## Results

### ABE-correction of *CDKL5*-R550* mutation in HEK293T cells

**Fig. 1:**
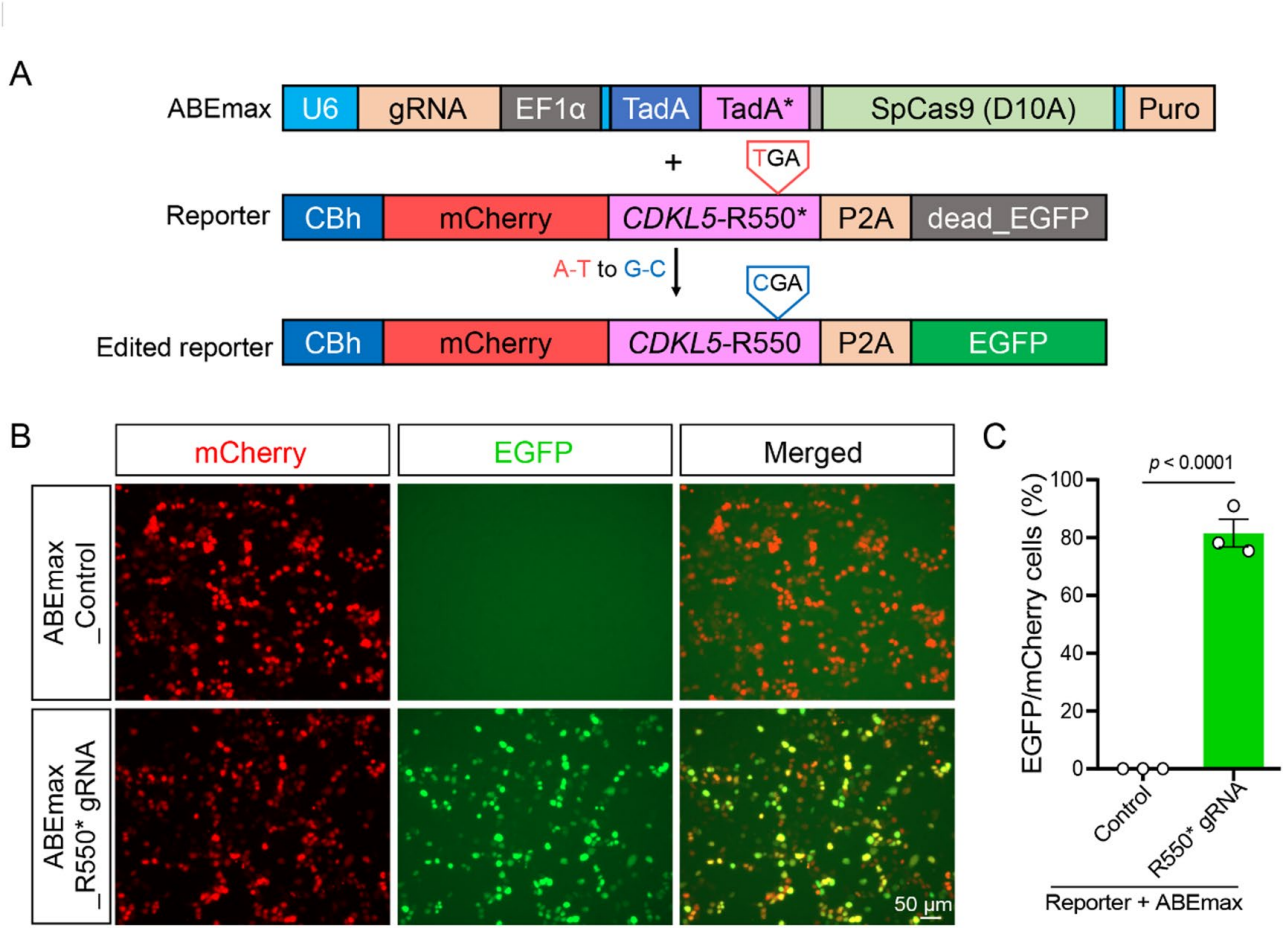
Design and validation of ABE gene editing system. (**A**) Schematic design for validating the efficacy of the ABE system for correcting a pathogenic nonsense mutation (*CDKL5*-R550*). The reporter consists of a CBh promoter driving expression of an mCherry cassette followed by a stop codon (TGA) cloned from *CDKL5* c.1648 C > T variant, and a EGFP cassette. Targeting the reporter with an ABEmax results in A-to-G conversion on the antisense DNA strand, removing the stop codon and allowing for the co-expression of EGFP reporter with mCherry. (**B**) Representative fluorescent images of ABE reporter system. Reporter plasmid was transfected with ABEmax_gR550* or ABEmax_gControl plasmid respectively in HEK 293T. Scale bar: 50 μm. (**C**) The percentage of mCherry vs. EGFP. Editing efficiency of ABEmax for *CDKL5*-R550* mutation is reflected using reporter system. Data are shown as mean ± SEM. Unpaired *t*-test is performed (*n* = 3).

To establish the gene correction strategy, we developed an ABE reporter system by cloning the *CDKL5*-R550* mutation sequence into the CBh-mCherry-target-EGFP vector as a Reporter^26^. The original target sequence was replaced with a 51-bp sequence containing the *CDKL5*-R550* mutation (Fig. 1A). In this system, cells transfected with the Reporter plasmid and ABEmax_Control expressed only mCherry fluorescence due to premature translation termination (Fig. 1B). However, successful ABE-correction by ABEmax_gR550* restored EGFP expression, resulting in co-expression of mCherry and EGFP (Fig. 1B). Editing efficiency was estimated by calculating the percentage of EGFP-positive cells in the population of mCherry-positive cells, reaching near 80% correction in the ABE reporter system (Fig. 1C). These findings demonstrate that the *CDKL5*-R550* mutation is amenable to ABE-correction and highlight the utility of this reporter system for evaluating base editing efficiency in other nonsense mutations.

### ABE-correction of *CDKL5*-R550* mutation in patient-derived iPSCs

**Fig. 2:**
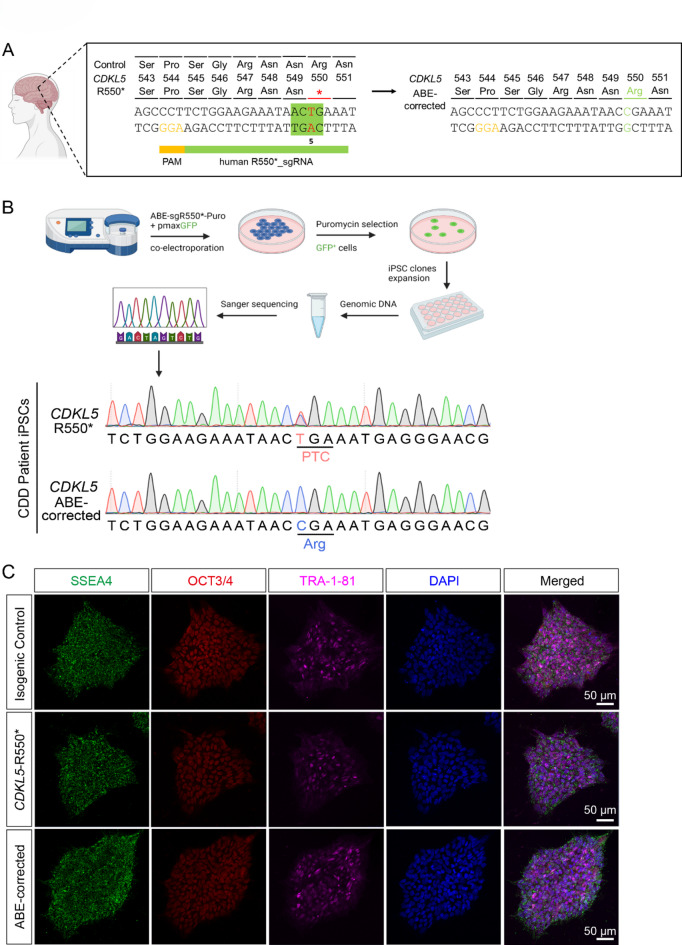
Precise correction of *CDKL5*-R550* mutation by ABE in iPSCs. (**A**) A candidate gRNA, hR550*_sgRNA, for base editing to correct the *CDKL5* c.1648 C > T (p.R550*) nonsense mutation. The guide sequence was marked by a green bar and the PAM (protospacer adjacent motif) site was in yellow. Base editing could convert the premature termination codon back to the naturally positive-charged arginine, restoring normal function of the CDKL5 kinase. The green-highlighted nucleotides refer to the ABE gene editing window, and the red nucleotide refers to the c.1648 C > T mutation. (**B**) Schematic flow for generating ABE-corrected iPSCs. Single-cell dissociated iPSCs are electroporated with ABEmax_gR550* with EGFP plasmids. After electroporation, cells are treated with puromycin for 2 days to remove non-transfected cells, and the EGFP positive cells are recovered and expanded in normal E8 medium. ABE-corrected iPSCs are identified after Sanger sequencing. Representative Sanger sequencing results of CDD patient-derived iPSCs after ABE gene editing, CDD patient iPSCs (upper panel, heterozygous), ABE-corrected iPSCs (lower panel). Schematic images were drawn by BioRender (https://www.biorender.com/). (**C**) Representative images of iPSC colonies after ABE-correction co-stained with pluripotency markers including SSEA4 (green), OCT3/4 (red) and TRA-1-81 (purple). Scale bar: 50 μm.

To test whether ABE could functionally correct the *CDKL5*-R550* mutation, we applied the approach to a heterozygous CDD patient-derived iPSC line carrying the *CDKL5*-R550* mutation (Fig. 2A)^25^. The same human R550*_gRNA used in the ABE reporter system was utilized for gene correction. Given that the *CDKL5*-R550* mutation will lead to the absence of the protein and cause the disease^17,18^, compared to its isogenic Control iN cells (Supplementary Fig. 1), we hypothesized that ABE-correction would restore the CDKL5 protein level and rescue the CDD-associated neuronal deficits. To test this, we electroporated ABEmax_gR550* plasmid mixed with 10% EGFP plasmid to the *CDKL5*-R550* iPSCs, replated the cells on mouse embryonic fibroblasts, and selected ABE-corrected iPSCs via puromycin resistance (Fig. 2B). Sanger sequencing confirmed precise correction in 2 iPSC colonies out of 24 with neat cytosine signal (lower panel in Fig. 2B) and no bystander edits in the editing window, compared to the dual signals in the original patient iPSCs (heterozygous, upper panel in Fig. 2B). After ABE-correction, iPSC line corrected by ABE displayed a normal karyotype (Supplementary Fig. 2). Furthermore, we checked the pluripotency of ABE-corrected iPSCs, as indicated by the expression of pluripotency markers via immunostaining including OCT4, SSEA4, TRA-1-81. (Fig. 2C), indicating that ABE-correction didn’t impact the pluripotent potential of the iPSCs.

### Off-target analysis following ABE-correction in iPSCs

**Fig. 3:**
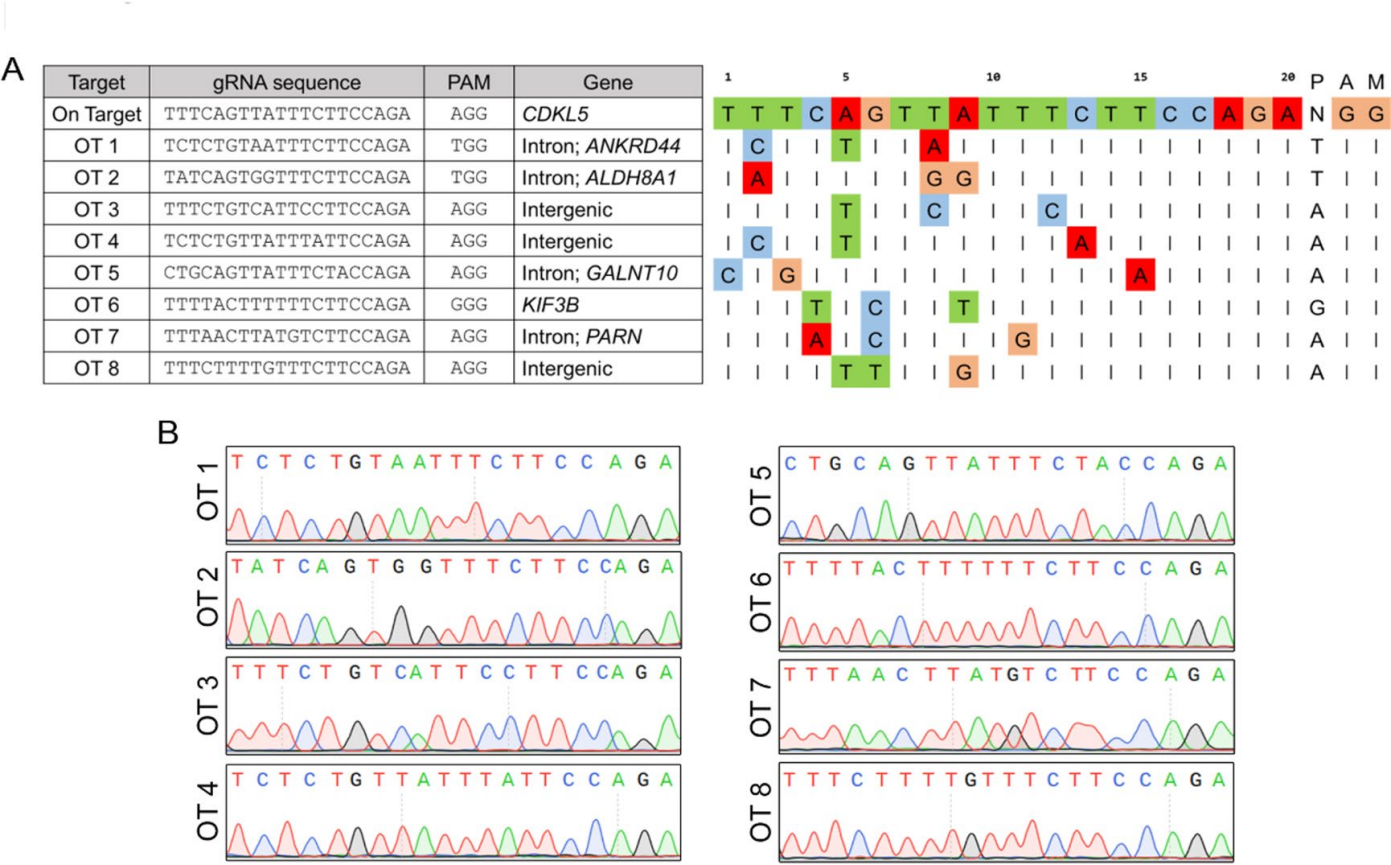
Off-target analysis following ABE-correction in iPSCs. (**A**) Genomic loci of eight candidate off-target (OT) sites (left) and alignment of eight candidate off-target sequence to the hR550*_gRNA sequence (right). Nucleotides that match the protospacer are indicated with a vertical dash, and nucleotides that differences are shown for each site. (**B**) Sanger sequencing results of the eight candidate OT sites.

Although base editors offer efficient and precise base conversions, unintended off-target (OT) modifications remain a concern for clinical applications^27^. Previous studies have shown that ABEs induce very low sgRNA-independent deamination of DNA, prompting us to focus on sgRNA-dependent off-target effects^28^. Using the CRISPOR predictive algorithm, we identified eight candidate off-target sites in the human genome (Fig. 3A). These regions were PCR-amplified from ABE-corrected iPSCs and analysed via Sanger sequencing. No unintended base edits were detected (Fig. 3B), indicating that ABE-correction of the *CDKL5* mutation in iPSCs is precise with low incidence of off-target events.

### Functional rescue of *CDKL5*-R550* mutation in patient-derived neurons

**Fig. 4:**
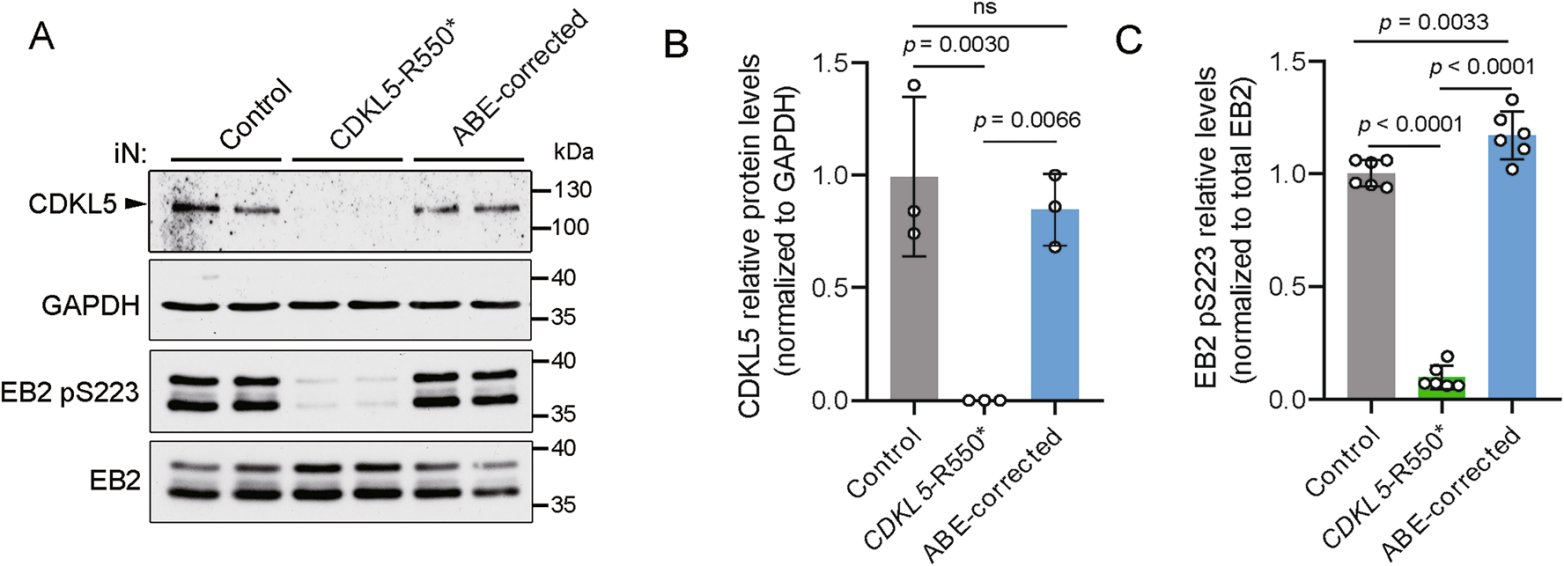
Restoration of CDKL5 expression and CDKL5-dependent signalling pathways. (**A**) Western blot showing the expression of CDKL5, total EB2 and EB2 pS223 in Control, *CDKL5*-R550* and ABE-corrected DIV28 iN cells. CDKL5, EB2 pS223, and EB2 were detected from the same gel. GAPDH was probed from another gel with same loading. CDKL5 is normalized to GAPDH and EB2 pS223 is normalized to total EB2. The unprocessed scans of representative western blots are included in Supplementary Fig. 3. Membranes were cut into strips prior to antibody incubation, therefore, images of all individual strips and all replicates are provided in Supplementary Fig. 5. Quantitative data of CDKL5 (**B**) and EB2 pS223 (**C**) levels are analysed by one-way ANOVA Dunnett’s multiple comparisons test, *n* = 3 for CDKL5 and *n* = 6 for EB2 pS223. Data are shown as mean ± SEM.

Previous studies have demonstrated that CDD patient-derived iPSC-induced neurons serve as a robust model for studying disease pathogenesis in vitro^21^. To evaluate whether ABE-correction restores CDKL5 function, we differentiated iPSCs into cortical neurons using an Ngn2-induced protocols^29,30^. Consistent with previous findings, CDKL5 protein was absent in *CDKL5*-R550* iN cells^17^, whereas ABE correction successfully restored CDKL5 expression to levels comparable to Control iN cells (Fig. 4A and B). The phosphorylation of EB2 Ser222 (mouse) has been used as a surrogate to assess the therapeutic effect of CDKL5-dependent signalling pathways^9^. Consistent with prior studies, phosphorylation of EB2 at Ser223 (human) was significantly reduced in *CDKL5*-R550* iN cells (Fig. 4A and C)^31^, whereas ABE-correction restored EB2 Ser223 phosphorylation to nearly Control levels, further demonstrating the functional rescue of CDKL5-denpendent signalling pathways by ABE-correction (Fig. 4A and C).

**Fig. 5:**
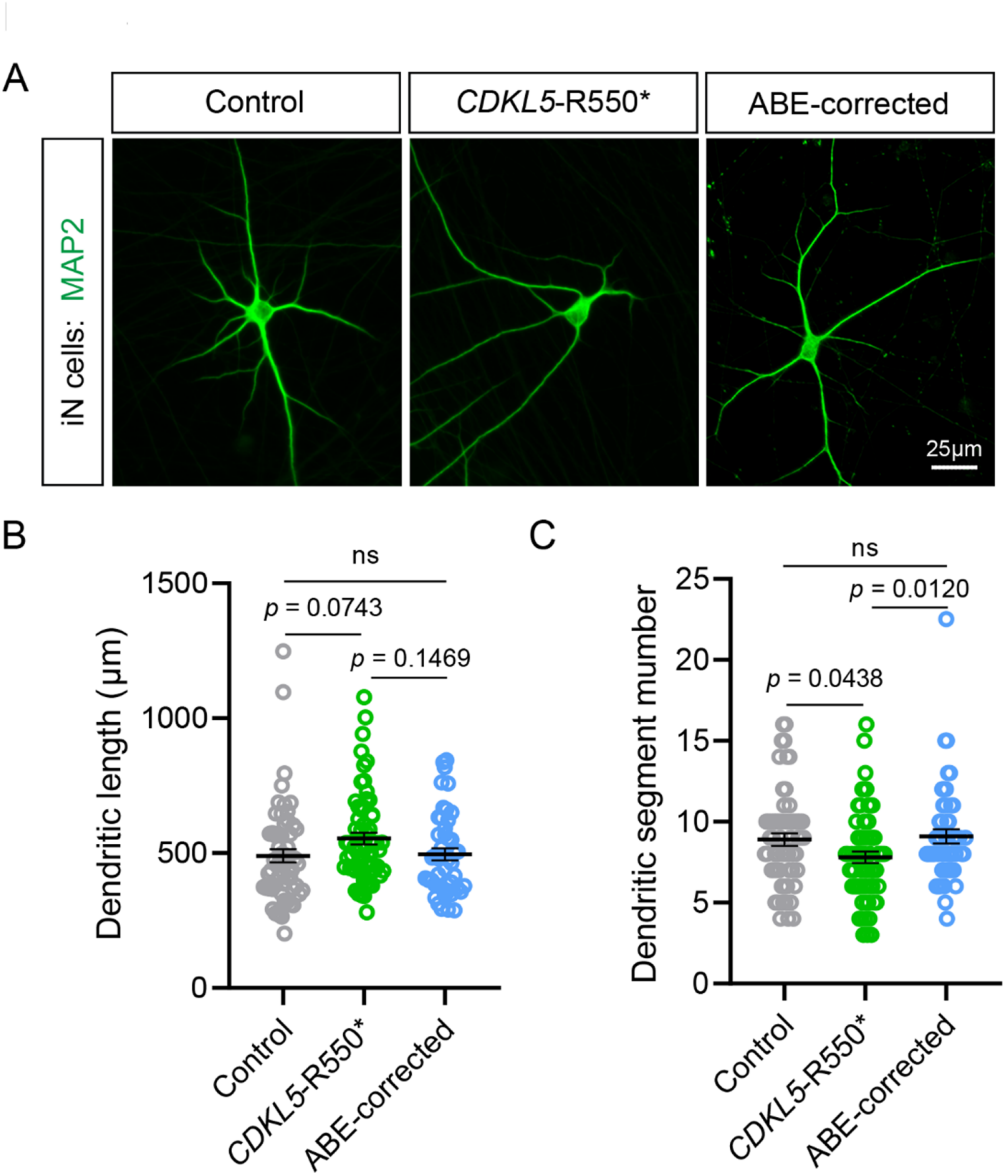
ABE-correction rescued dendritic deficits in *CDKL5*-R550* iN cells. (**A**) Immunofluorescence staining of Control, *CDKL5*-R550* and ABE-corrected iN cells. Scale bar: 25 μm. Sholl analysis is performed to analyse the dendritic length (**B**), and dendritic segment number (**C**) on Control (grey), *CDKL5*-R550* (green) and ABE-corrected (blue) DIV28 iN cells. Data are shown as mean ± SEM. One-way ANOVA Dunnett’s multiple comparisons test is performed for dendritic length and dendritic segment number (Control, *n* = 59; *CDKL5*-R550*, *n* = 62; ABE-corrected, *n* = 46).

To further validate the therapeutic potential of ABE correction, we analysed dendritic morphology. *CDKL5*-R550* iN cells exhibited significantly increased dendritic length compared to Controls, consistent with previous findings by Negraes et al.^24^ (Fig. 5A and B). Notably, this abnormality was normalized in ABE-corrected iN cells (Fig. 5B). Additionally, the dendritic segment number was also significantly reduced in *CDKL5*-R550* iN cells compared to Controls, while no significant difference was observed between Control and ABE-corrected iN cells (Fig. 5C). These findings provide additional evidence supporting the functional restoration of neuronal morphology through base editing.

### Normalization of neuronal gene expression by ABE

**Fig. 6:**
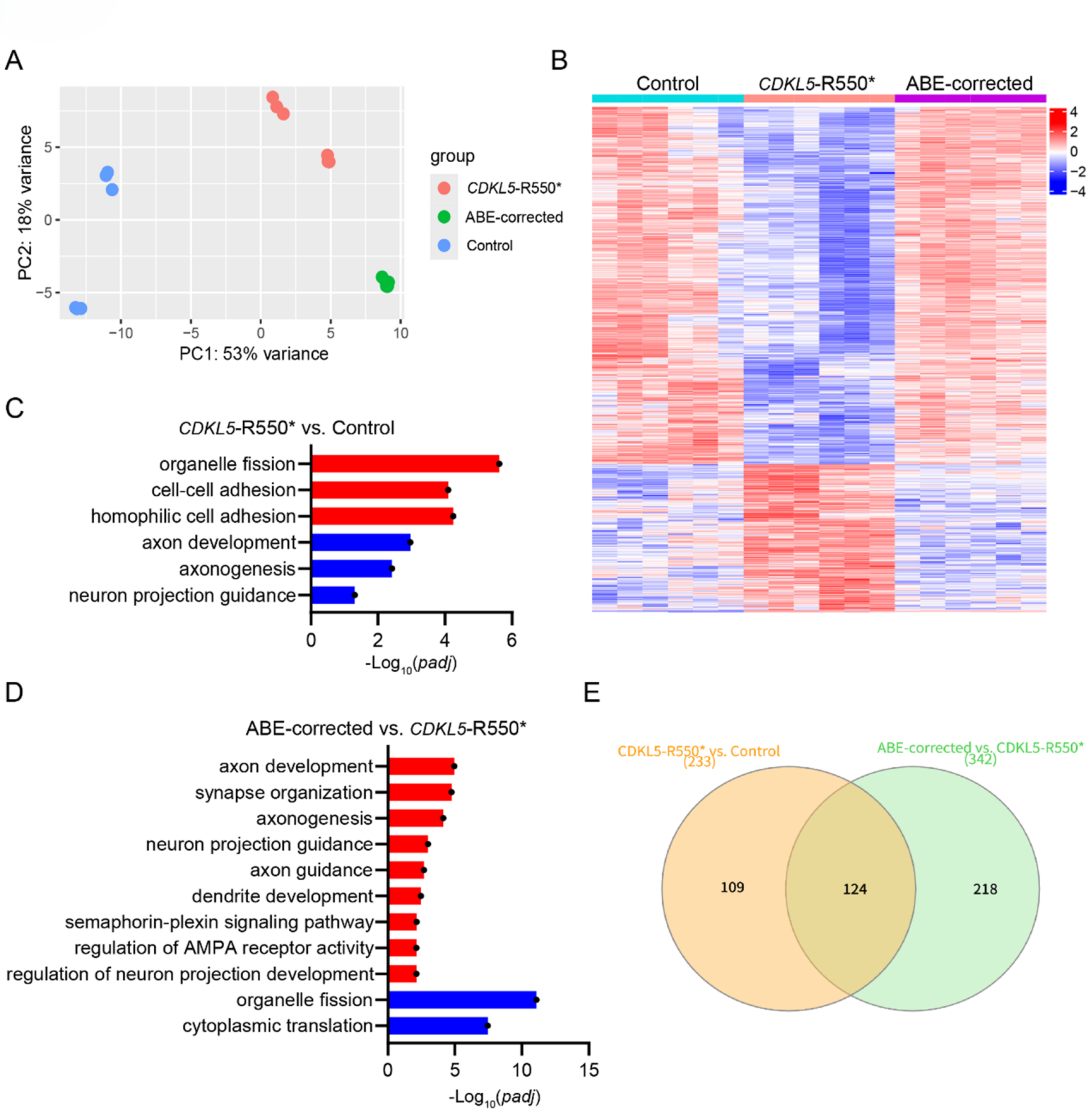
ABE correction normalized transcriptional expression in *CDKL5*-R550* iN cells. (**A**) PCA plot showing RNA-seq clusters of Control, *CDKL5*-R550* and ABE-corrected iN cells. (**B**) Heatmap showing the expression of differentially regulated genes in Control, *CDKL5*-R550* and ABE-corrected iN cells. (**C**) GO terms associated with the up-regulated and down-regulated genes in *CDKL5*-R550* compared to Control iN cells. (**D**) GO terms associated with the up-regulated and down-regulated genes in ABE-corrected compared to *CDKL5*-R550* iN cells. (**E**) Venn diagram comparing GO terms of *CDKL5*-R550*_vs._Control and GO terms of ABE-corrected_vs._*CDKL5*-R550*. Venn diagram was drawn by InteractiVenn (https://www.interactivenn.net/).

To determine whether ABE-correction could restore normal gene expression in *CDKL5*-R550* iN cells, we performed RNA-seq analysis on isogenic Control, *CDKL5*-R550* and ABE-corrected DIV28 iN cells. The PCA plot showed the separation of three groups, but ABE-corrected and Control iNs were not closely clustered (Fig. 6A). However, as shown in the heatmap (Fig. 6B), transcriptional alterations between Control and *CDKL5*-R550* iN cells were widely normalized after ABE-correction. These changes included the up-regulation of genes associated with organelle fission and cell-cell adhesion, as well as the down-regulation of genes involved in axon development and neuron projection (Fig. 6C) in Gene Ontology (GO) term enrichment. Notably, ABE-correction normalized the gene expression profile of *CDKL5*-R550* iN cells, as evidenced by the down-regulation of genes that were previously up-regulated and the up-regulation of neuronal genes that were initially down-regulated in the mutant iN cells (Fig. 6D). Particularly, we observed the up-regulated genes were enriched in pathways related to axon development, synapse organization, dendrite development, and regulation of AMPA receptor activity (Fig. 6D). The transcriptome restoration aligns with previous findings in this study. Furthermore, in the Venn diagram of these GO terms between groups (Fig. 6E), more than half of the GO terms in the group of *CDKL5*-R550*_vs._Control were shared with the group of ABE-corrected_vs._*CDKL5*-R550*. However, there are still unique GO terms between the groups, and it requires further investigation for the transcriptomic changes after ABE-correction. In addition, we also validated the gene expression by quantitative real-time PCR, which confirmed that the abnormally up-regulated gene, for example, *EPHA7* was down-regulated in ABE-corrected iN cells (Supplementary Fig. 4). This result further supports the conclusion that ABE gene editing effectively normalizes gene expression, highlighting its potential to restore neuronal function and mitigate the molecular pathologies of CDD.

## Discussion

In this study, we focused on a human pathogenic nonsense point mutation *CDKL5* c.1648 C > T (p.R550*) and performed a proof-of-concept study to demonstrate that ABE efficiently corrected the C-to-T mutation and yielded therapeutic effects in iN cells. We assessed the therapeutic effects from three aspects of CDKL5 disorder. On the one hand, ABE-correction restored *CDKL5* expression with similar protein level to the endogenous in isogenic Control, and this is the fundamental basis for the rescue. This is also important to the treatment of CDD, because some studies reported that CDKL5 duplication can also lead to macrocephaly and learning disabilities, suggesting that tight regulation of CDKL5 levels is essential to maintain the normal function in the central nervous system^12,32^. Consequently, this restored endogenous expression of CDKL5 in ABE-corrected iN cells directly led to the reactivation of CDKL5-dependent signalling pathways, and partially rescued some neuronal morphological deficits in iN cells. This result is in line with our hypothesis that correction of the *CDKL5* DNA point mutations can restore the biological function of CDKL5 protein, suggesting therapeutic effects of ABE. While the observed dendritic changes were less pronounced than the biochemical phenotypes, likely due to limitations of the Ngn2-induced neuron model, because this method quickly skipped the neural progenitor cells (NPCs) stage in the differentiation, and the neurons may not be mature enough with only four weeks induction^33^. So, the assessed therapeutic parameters related to neuronal differentiation and maturation are limited in this proof-of-concept study. Additionally, CDD-derived NPCs were reported to have proliferation defects^24^. Future work will also focus on NPC induction and improvement of neuronal differentiation to analyse the rescue of the proliferation and synapse defects after ABE-correction. Altogether, these morphological data collectively suggest that the immature dendritic architecture of *CDKL5*-R550* iN cells was partially rescued by ABE-correction.

To have a comprehensive understanding of the therapeutic effects on the differentiated neurons, we performed transcriptomic analysis of Control, *CDKL5*-R550* and ABE-corrected iN cells, and the results revealed a normalization of many differentially expressed genes and pathways following ABE-correction. Notably, genes involved in axonogenesis and neuron projection guidance, which were downregulated in *CDKL5*-R550* iN cells, were restored to Control levels after ABE-correction. Similarly, several abnormally upregulated genes in *CDKL5*-R550* iN cells were downregulated following ABE-correction. The transcriptomic normalization can also be aligned to the morphological phenotypes, for example, *EPHA7* expression was normalized in ABE-corrected iN cells. Overexpression of *Epha7* has been shown to inhibit dendritic growth and spine formation in primary neurons, leading to shorter dendrites and fewer dendritic branches compared to controls^34,35^. The *EPHA7* qPCR validation supports the amelioration of the morphological deficits of dendritic length and segment number following ABE-correction.

In this study, we provided therapeutic evidence from ABE-corrected iPSCs. As a future direction, it will be informative to assess if ABE can directly rescue the CDD-related deficits on the differentiated neurons and brain organoids. Additionally, the phenotypes of NPCs and iN cells can be analysed for proliferation, viability, synaptic formation, synaptic activation and CDKL5-dependent signalling pathways. Intriguingly, the PCA blot and Venn diagram of GO terms in Fig. 6 suggested that there are still some differences between ABE-corrected and Control iN cells. This partial transcriptome rescue is similar to previous studies in iPSC-derived neuronal models. For instance, dendritic outgrowth is impaired in *SERPINI1* knock-in (KI) iN cells and partially rescued by ABE treatment. The neuronal morphology of ABE-corrected iN cells lies between WT and KI^36^. The observed transcriptional differences between ABE-corrected and Control iN cells in our study, despite normalization, may partially stem from the inherent clonal heterogeneity in iPSC lines or variations induced by Ngn2 induction protocol^37,38^.

As with all therapeutic applications of genome editing agents, potential off-target editing on both DNA^16,28^ and RNA^39^ needs to be taken into account. Especially, for AAV-delivered base editors, these viruses can sustain long-term expression, increasing the potential of off-target editing. In the previous study, ABEs are reported to induce very low sg-independent deamination of DNA^28^, so allowed us to focus on the sg-dependent off-targets by testing the CRISPOR-predicted off-target sites. In our study, the electroporated ABEmax plasmid only sustained a transient expression and degraded in the following days, therefore, it decreased the off-target editing potential on DNA and RNA. Screening for off-target effects with whole-genome and transcriptome-wide sequencing isn’t standardized in this field, but it offers better understanding and delineation of these effects^40^. The improvement of high efficiency, low off-target and wide-range CRIPSR-based gene therapy is still on-going in this field.

## Conclusion

In summary, our study demonstrates that ABE serves as an efficient and precise tool for correcting point mutations in *CDKL5*. The therapeutic efficacy of this approach was evidenced by comprehensive biochemical, morphological, and transcriptomic analyses, which collectively demonstrated functional restoration of CDKL5 and CDKL5-dependent signalling pathways in iN cells. While further therapeutic validation and safety assessments in CDD mouse models, differentiated iN cells and brain organoids remain essential, this ABE-correction strategy holds great potential for treating CDD and may be broadly applicable to other NDDs caused by pathogenic point mutations.

## Materials and methods

### iPSC culture

Two iPSC lines derived from the same female CDD patient, both carrying a heterozygous *CDKL5* c.1648 C > T mutation but differing in their X-chromosome inactivation status—resulting in the expression of either the mutant or wild-type *CDKL5* transcript—were obtained from the Coriell Institute (*CDKL5*-R550*: OR00005; Control: OR00006). This design ensured isogenic comparability between the control and mutant lines, allowing for a precise evaluation of the mutation-specific effects^25^. iPSCs were cultured on Geltrex (Thermo Fisher Scientific, A1413302) in Essential 8 medium (E8, Thermo Fisher Scientific, A1517001) and passaged upon reaching 80% confluency using 0.05 µM UltraPure EDTA solution (Thermo Fisher Scientific, 15575020).

### Plasmids for *CDKL5*-R550* correction

To establish the ABE system for correcting *CDKL5*-R550* mutation, all-in-one U6-empty-ABEmax-puro vector was reconstructed. ABEmax was PCR-amplified from the pCMV-ABEmax-P2A-GFP plasmid (Addgene #112101) and ligated into the modified pSpCas9(BB)-2A-Puro vector (Addgene #48139) under the EF1α promoter. The *CDKL5*-R550* gDNA (5’-TTTCAGTTATTTCTTCCAGA-3’) was annealed and ligated into the U6-empty-ABEmax-puro vector. Additionally, a 51 base-pair sequence containing the *CDKL5*-R550* mutation (5’-CTCAGCCCTTCTGGAAGAAATAAC**T**GAAATGAGGGAACGCTGGACTCACGT-3’) was synthesized and cloned into the homemade CBh-mCherry-target-EGFP vector as a base editing reporter. Successful correction of the in-frame nonsense mutation in the target sequence restores EGFP expression, serving as a readout for efficient base editing.

### Cell culture and transfection

HEK293T cells were cultured in DMEM (Thermo Fisher Scientific, 10565042) supplemented with 10% FBS (Thermo Fisher Scientific, 26010074) and Penicillin-Streptomycin (Thermo Fisher Scientific, 15140122). Cells were replated onto 6-well plate when reaching 80% confluency, and we transfected HEK293T cells using Lipofectamine 3000 transfection kit (Thermo Fisher Scientific, L3000015) according to the manufacturer’s protocol with plasmids: Reporter (0.4 µg) + ABEmax_Control (1.6 µg) or Reporter (0.4 µg) + ABEmax_R550* gRNA (1.6 µg) in each well of 6 well plate. After 48 h, the cells were imaged through the fluorescence microscope (ZEISS Axio Observer) using 20× objective with three independent replicates. We used FIJI software Cell Counter plug-in to count the number of EGFP positive cells and mCherry positive cells respectively in each group, and then calculated the percentage of EGFP/mCherry to reflect the editing efficiency.

### Nucleofection and genotyping

Prior to nucleofection, iPSCs were pretreated with 10 µM ROCK inhibitor (Sigma Aldrich, Y0503-5MG) for 1 h. Single-cell suspensions were prepared using StemPro™ Accutase (Thermo Fisher Scientific, A1110501). For ABE-correction, iPSCs were electroporated with 5 µg ABEmax and 0.5 µg EGFP using the Human Stem Cell Nucleofector 1 (Lonza, VPH-5012) and Nucleofector 2b Device according to the manufacturer’s protocol. Cells were replated onto mouse embryonic fibroblast (MEF) feeders (Thermo Fisher Scientific, A24903) and treated with 0.5–1 µg/mL puromycin (Thermo Fisher Scientific, A1113803) 24 h post-nucleofection. The iPSC colonies were expanded and genotyped according to half-colony strategy^41^. We used tips to scrape and pipette half colony of the iPSCs for expansion, and the other half for genotyping under the microscopy. The iPSC genomic DNA of the half-colony was extracted using QuickExtract DNA extraction solution (Epicentre, QE09050). PCR primers targeting exon 12 of *CDKL5* were designed in Supplementary Table 2 for genotyping. PCR reactions were performed using DreamTaq PCR Master Mixes (2X) (Thermo Fisher Scientific, F1802). PCR products were purified by FavorPrep™ Gel/PCR Purification Kit (FAVORGEN, FAGCK 001–1) according to the manufacturer’s protocol, and were analysed via Sanger sequencing at BGI.

### Differentiation of iPSCs into neurons

Differentiation of iPSCs into neurons was performed as previously described^29,30^ with modifications. Briefly, iPSCs were plated on Geltrex-coated 6-well plates and infected with lentivirus containing rtTA and Ngn2-GFP-Puro vectors in E8 medium supplemented with 10 µM ROCK inhibitor. On day 0, Ngn2 expression was induced with 2 µg/mL doxycycline (Sigma Aldrich, D9891-1G). On day 1, non-infected cells were eliminated by puromycin selection (1 µg/mL). On day 2, cells were switched to N2 medium (DMEM/F12 [Thermo Fisher Scientific, 10565042], 1× N2 supplement [Thermo Fisher Scientific, 17502048] and 1× NEAA [Thermo Fisher Scientific, 11140050]) containing 2 µg/mL doxycycline and 1 µg/mL puromycin. On day 3, induced neurons were dissociated with Accutase and replated at densities of 2.0 × 10⁶ cells/well (6-well plates) or 1.0 × 10⁵ cells/coverslip (24-well plates; SPL Life Sciences, 20012) on laminin-coated plate (Sigma Aldrich, L2020-1MG) in B27 medium (Neurobasal [Thermo Fisher Scientific, 21103049], 1× B27 supplement [Thermo Fisher Scientific, 17504044] and 1× GlutaMAX [Thermo Fisher Scientific, 35050061]) containing 2 µg/mL doxycycline and 1 µg/mL puromycin. From day 6–7, the medium was replaced with B27 medium containing 1 µg/mL doxycycline, 50 nM AraC (Sigma Aldrich, C6645), 0.2 µg/mL laminin, 10 ng/mL BDNF (PeproTech, 450-02) and 10 ng/mL GDNF (PeproTech, 450 - 10). Thereafter, half of the medium was refreshed twice a week until day 28.

### Protein extraction and western blot

Cells were lysed in RIPA buffer containing protease and phosphatase inhibitors, then centrifuged at 14,000×rpm for 15 min at 4 °C. Supernatants were collected, and protein concentration was measured using the Bradford Protein Assay Kit (Bio-Rad, 5000201). Equal amounts of protein were resolved via 10% SDS-PAGE and transferred to nitrocellulose membranes. Following transfer, full-length membranes were cut into two strips according to the expected molecular weight prior to incubation with primary antibodies. Membranes were blocked with 5% milk in TBST and incubated with primary antibodies (Supplementary Table 1) for CDKL5 (1 h, room temperature) or GAPDH, EB2 pS222, and total EB2 (overnight, 4 °C), followed by HRP-conjugated secondary antibody incubation for 1 h at room temperature. Signals were detected using SuperSignal WestPico Chemiluminescent Substrate (Thermo Fisher Scientific, 34577) and Super RX medical X-ray films (Fujifilm, 47410).

### Immunofluorescence staining, image acquisition and analysis

Cells were fixed with 4% formaldehyde solution (Sigma Aldrich, 47608) at room temperature for 15 min, and incubated in blocking buffer (DPBS, 0.1% Triton-X 100, 4% goat serum and 1% BSA) for 30 min. Primary antibodies were diluted in blocking buffer and incubated at 4 ℃ overnight, followed by DPBS washes and incubation with secondary antibodies and DAPI at room temperature for 1 h. Cells were washed 3 times and mounted with Fluoromount-G Mounting Medium (Thermo Fisher Scientific, 00-4958-02). Molecular Devices ImageXpress Micro Confocal High Content Screening System (iN cells) and Zeiss LSM 900 Confocal microscopy (iPSCs) were used to acquire fluorescent images using a 20× objective. The images from the same experiments were acquired using same acquisition settings. And results for three independent experiments were put together for analysis. For the dendritic length and segment number, we performed sholl analysis and measurements using FIJI SNT plug-in^42^.

### RNA extraction, genomic DNA extraction and quantitative real-time PCR

Genomic DNA of the iPSCs were extracted using Rapid Animal Genomic DNA Isolation Kit (Sangon Biotech, B518221). And total RNA from iN cells were extracted using TRIzol (Thermo Fisher Scientific, 15596026CN) according to the manufacturer’s protocols. RNA concentration and quality were assessed using a NanoDrop 2000. First-strand cDNA was generated through Hifair II 1st Strand cDNA Synthesis SuperMix (YEASEN, 11120ES60). Quantitative real-time PCR was performed using Power SYBR Green PCR master mix (Thermo Fisher Scientific, 4368577). The gene expression level of mRNA was normalized to *ACTIN*. 

### RNA sequencing and data analysis

RNA samples of 4-week differentiated neurons were used for mRNA purification by oligo-dT beads and fragmentation. First-strand cDNA library construction and transcriptome profiles were generated by Novogene (Beijing) on Illumina NovaSeq 6000 platform. Briefly, FastQC was used to estimate the quality of the files. Read mapping, counting, and differential gene expression analysis of these neurons were performed using HISAT2^43^, FeatureCounts^44^ and DESeq2^45^ respectively. The criteria for the differentially expressed genes are: |log2(FoldChange)| > 0.25 and *padj* < 0.05. GO analysis was performed using clusterProfiler^46^, and selective GO terms were shown.

### Off-target analysis

The top eight sg-dependent off-target sites of human R550*_sgRNA of ABEmax were predicted using CRISPOR Version 5.2 (https://crispor.gi.ucsc.edu/)^47^. Off-target primers (see Supplementary Table 3) were used for PCR amplification of genomic DNA from ABE-corrected iPSCs, followed by Sanger sequencing at BGI.

### Statistical analysis

Data are presented as mean ± SEM. Statistical significance was assessed using Student’s *t*-test, or one-way ANOVA as appropriate. All experiments were performed with at least three independent replicates.

## Supplementary Information

Below is the link to the electronic supplementary material.

Supplementary information 2: DEGs_three_groups. Control (*n* = 6), CDKL5-R550* (*n* = 6) and ABE-corrected (*n* = 6).

Supplementary Material 2

## Data Availability

The datasets generated and analysed during the current study are available in the Gene Expression Omnibus (GEO) repository (Accession No: GSE325168).
