# Supplementary material for: Base editing restores CDKL5 expression and rescues neuronal deficits in a patient-derived model of CDKL5 deficiency disorder
Source: Sci Rep. 2026 Apr 10;16:16151. doi: 10.1038/s41598-026-48097-y (PMC13201562; doi:10.1038/s41598-026-48097-y)
Supplement: Supplementary file 2 — Supplementary Material 2 [file 41598_2026_48097_MOESM2_ESM.pdf]

Base editing restores CDKL5 expression and rescues neuronal deficits in a patient-derived model of CDKL5 deficiency disorder

Yue Chai[1,2], Yao Zhu[1], Jiayi Zhu[3], Mingfeng Guan[2], Zhongyu Zheng[1], Yu Chen[2,3], Hayley Wing Sum Tsang[4], Tao Ye[2,3*], Jacque Pak Kan Ip[1,5,6*]

[1]School of Biomedical Sciences, The Chinese University of Hong Kong, Hong Kong SAR, China

[2]Shenzhen Key Laboratory of Translational Research for Brain Diseases, The Brain Cognition and Brain Disease Institute, Shenzhen Institutes of Advanced Technology, Chinese Academy of Sciences, Shenzhen-Hong Kong Institute of Brain Science—Shenzhen Fundamental Research Institutions, Shenzhen, China

[3]Guangdong Provincial Key Laboratory of Brain Science, Disease and Drug Development, HKUST Shenzhen Research Institute, Shenzhen, China

[4]Division of Life Science, The Hong Kong University of Science and Technology, Hong Kong SAR, China

[5]CUHK Shenzhen Research Institute, The Chinese University of Hong Kong, Shenzhen, China

[6]Gerald Choa Neuroscience Institute, The Chinese University of Hong Kong, Hong Kong SAR, China

Supplementary Table 1. Antibodies and concentrations used for Western blot and Immunofluorescence staining.

Antibody/Target	Manufacturer	Catalog #	Dilution
CDKL5 (360-650 human)	MRC Dundee	S957D	1:500
CDKL5 Antibody (D-12)	Santa Cruz Biotechnology	sc-376314	1:500
EB2 pS222 antibody	Covalab	pab01032-P	1:2000
Anti-EB2 antibody [KT52]	Abcam	ab45767	1:2000
GAPDH Monoclonal Antibody (6C5)	Invitrogen	AM4300	1:3000
Oct3/4 Antibody (C-10)	Santa Cruz Biotechnology	sc-5279	1:200
SSEA4 Monoclonal Antibody (MC-813-70)	Thermo Fisher Scientific	41-4000	1:200
Anti-TRA-1-81 Antibody, clone TRA-1-81	Sigma Aldrich	MAB4381	1:200
Anti-MAP2 antibody-Neuronal Marker	Abcam	ab5392	1:5000
Goat anti-Mouse IgG3 Cross-Adsorbed Secondary Antibody, Alexa Fluor™ 488	Thermo Fisher Scientific	A-21151	1:500
Goat anti-Mouse IgG2b Cross-Adsorbed Secondary Antibody, Alexa Fluor™ 568	Thermo Fisher Scientific	A-21144	1:500
Goat anti-Mouse IgM (Heavy chain) Cross-Adsorbed Secondary Antibody, Alexa Fluor™ 633	Thermo Fisher Scientific	A-21046	1:500
Goat Anti-Chicken IgY H&L (Alexa Fluor® 568)	Thermo Fisher Scientific	ab175477	1:500

Supplementary Table 2. Primers for CDKL5-R550* genotyping and RNA-seq qPCR and genomic qPCR.

Genes	Primers	
CDKL5	Forward: 5’-AGTCCCTCCTACAGGACCAA-3’	Genotyping
	Reverse: 5’-CAAGCCCTTGGCTCTCACTT-3’	
EPHA7	Forward: 5’-AGCTACAGCTGTCTCCAGTG-3’	qPCR
	Reverse: 5’-TAACCACAGTGCCTTCTCCC-3’	
ACTIN	Forward: 5’-TTCTACAATGAGCTGCGTGTG-3’	
	Reverse: 5’-GGGGTGTTGAAGGTCTCAAA-3’	

Supplementary Table 3. Primers for top eight candidate off-target sites.

OT sites	Primers	Length
OT 1	Forward: 5'-ACTGAGTCCTTAGAAGTTTCCACA-3'	201
	Reverse: 5'-TATTGCAACTGGTCCTCCATGT-3'	
OT 2	Forward: 5'-AATTGCCATCAAGGGCCAGA-3'	289
	Reverse: 5'-CCAGTAGAGAGGGGAAGCCA-3'	
OT 3	Forward: 5'-GCCTTTTTCAGAACCAGCCA-3'	250
	Reverse: 5'-GGGGGCAATTCCAAACAACC-3'	
OT 4	Forward: 5'-GTGACATTACTTCAGGAACAGGA-3'	239
	Reverse: 5'-ACCCTTGAAGATTGGCTGAGTT-3'	
OT 5	Forward: 5'-CTGCTATCTGGGAAATCTGGC-3'	277
	Reverse: 5'-CCAGTGGAGAAATCAAGGAGTTAG-3'	
OT 6	Forward: 5'-ACCATGCATGAGACCAGATAGC-3'	284
	Reverse: 5'-ATCAAACCCCTTTGTTGACCA-3'	
OT 7	Forward: 5'-TCACAAGTAATTCTGAAAAGTCTCT-3'	200
	Reverse: 5'-TATTAATACAACACTTAGCACAGTC-3'	
OT 8	Forward: 5'-TCAGTTGAGACGTTACATGCAAAT-3'	244
	Reverse: 5'-TTGAAATGGCTCATGCTTGGAC-3'	

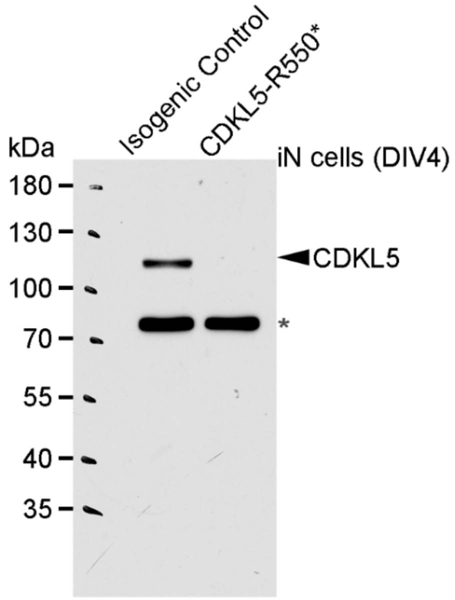

Supplementary Figure 1. CDKL5 expression in DIV4 differentiated *CDKL5*-R550* and isogenic Control iN cells. Asterisk in the 70 kDa shows the unspecific band.

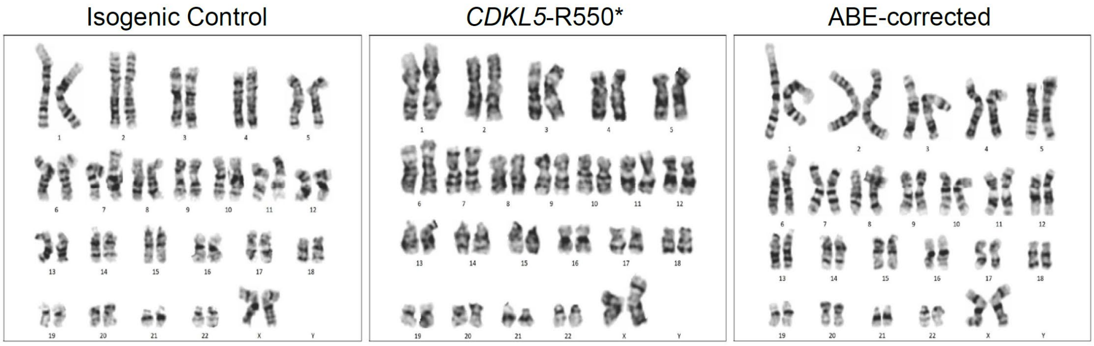

Supplementary Figure 2. Normal karyotype was observed in iPSCs after ABE correction.

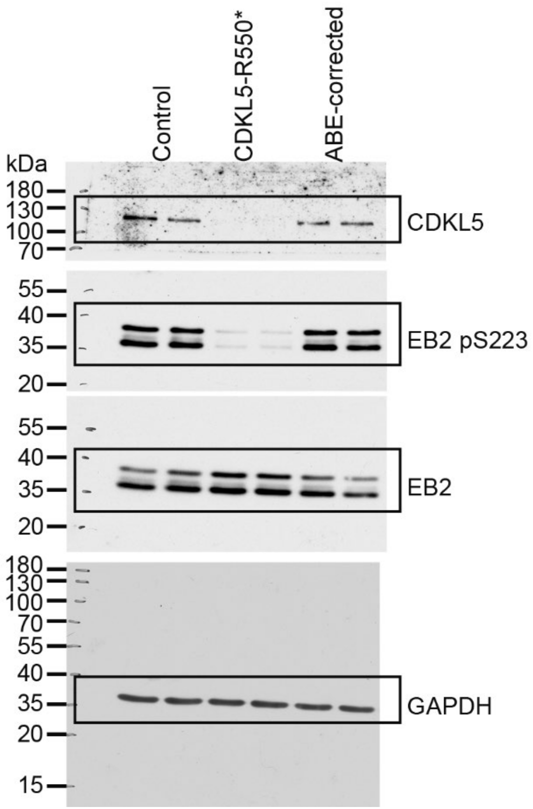

Supplementary Figure 3. The unprocessed original scans of western blots displayed on Figure 4A.

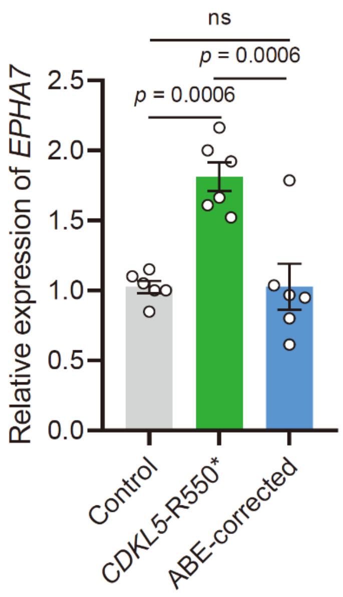

Supplementary Figure 4. qPCR validation of *EPHA7* in Control, *CDKL5*-R550* and ABE-corrected iN cells. The data was shown as mean ± SEM and analysed by one-way ANOVA Tukey's multiple comparisons test, n = 6.

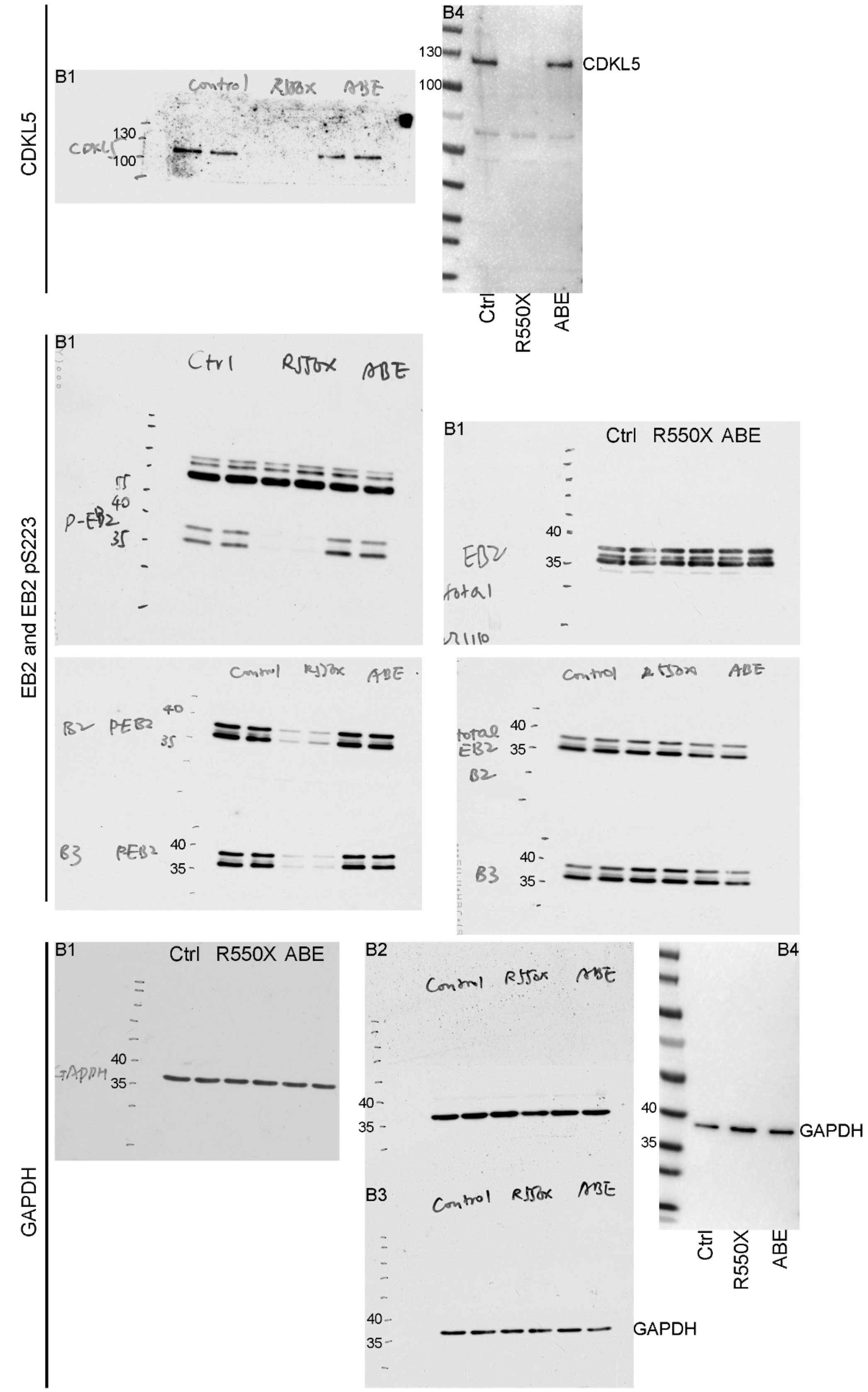

Supplementary Figure 5. Full scans for western blots.
